# Altered Resting-State Connectivity in College Students with Nonclinical Depressive Symptoms

**DOI:** 10.1371/journal.pone.0114603

**Published:** 2014-12-12

**Authors:** Xinhua Wei, Huicong Shen, Jiliang Ren, Xueli Li, Xiangdong Xu, Ruimeng Yang, Lisha Lai, Liang Chen, Jiani Hu, Wenhua Liu, Xinqing Jiang

**Affiliations:** 1 Department of Radiology, the affiliated Guangzhou first hospital, Guangzhou Medical University, Guangzhou, Guangdong, 510180, China; 2 Department of Radiology, Tiantan hospital, Capital Medical University, Beijing, 100050, China; 3 Department of Radiology, Wayne State University, Detroit, Michigan, 48202, United States of America; 4 Faculty of Health Management, Guangzhou Medical University, Guangzhou, Guangdong, 510180, China; Hangzhou Normal University, China

## Abstract

**Background:**

The underlying brain basis of nonclinical depressive symptoms (nCDSs) is largely unknown. Recently, the seed-based functional connectivity (FC) approach for analyzing resting-state fMRI (rs-fMRI) data has been increasingly used to explore the neural basis of depressive disorders. Other than common seed-based FC method using an *a priori* seed region, we conducted FC analysis based on regions with altered spontaneous activity revealed by the fractional amplitude of low-frequency fluctuations (fALFF) approach. The aim of the present study was to provide novel insight in the underlying mechanism of nCDSs in college students.

**Methodology/Principal Findings:**

A total number of 1105 college students were recruited to participant in a survey for assessing depressive symptoms. Subsequently, 17 individuals with nCDSs and 20 healthy controls (HCs) were enrolled to perform MR studies. Alternations of fALFF were identified in the right superior parietal lobule (SPL) and left lingual gyrus, both of which were used as ROIs for further FC analysis. With right SPL, compare with HCs, subjects with nCDSs showed reduced FCs in the bilateral dorsal lateral prefrontal cortex (DLPFC), left inferior frontal gurus (IFG), left premotor cortex (PMC), DMN network [i.e., bilateral precuneus, posterior cingulate cortex (PCC), right supramarginal gyrus (SMG), right parahippocampal gyrus (PHG), bilateral inferior temporal gurus (ITG)] and left cerebellum posterior lobe (CPL). In addition, increased FCs were observed between the left lingual gyrus and right fusiform gyrus as well as in the left precuneus.

**Conclusion/Significance:**

Our results indicate the abnormalities of spontaneous activity in the right SPL and left lingual gyrus and their corresponding dysfunction of the brain circuits might be related to the pathophysiology of nCDSs.

## Introduction

Major depressive disorder (MDD) is the single greatest cause of disability and morbidity in adolescence and young adulthood [1], and has been attracting increasing attention from researchers [2]. However, less attention has been given to non-clinical depressive symptoms (nCDSs) that present variable depressive symptoms but do not meet the diagnostic criteria of MDD [3]. Due to the undergoing and obvious social and physical changes, nCDSs have been found to be highly prevalent among college students worldwide [3]–[6], with the peak age of onset between 15 and 29 years of age [7]. Importantly, longstanding nCDSs associated with a great risk of developing depressive disorders [8]. However, to date, little knowledge has been gained with regard to the neural basis of nCDSs. Therefore, exploring the neural basis of nCDSs could be considerably important for understanding the pathophysiology of nCDSs.

Recently, a growing body of evidence has shown that the spontaneous brain activity measured with resting-state fMRI (rs-fMRI) reveals important information about brain function and its alteration. Low-frequency (0.01–0.08 Hz) oscillations (LFOs) in the resting-state are considered to be of physiological importance and are related to spontaneous neural activity of the brain [9], [10]. Over the past two decades, a number of brain regions with altered activity in the resting-state have been reported related to MDD, such as the prefrontal cortex [11], [12], anterior cingulate cortex [2], subgenual cingulate cortex [13], amygdala [2], [14], insular [15], thalamus [13], fusiform gyrus and the cerebellum [16]. In line with the intrinsic functional connection of spatially distributed brain regions, imbalanced activity and connectivity of mood-regulating circuit (prefrontal-limbic-thalamic regions) [17]–[21] are suggested as a possible mechanism of MDD. Furthermore, converging evidence indicates that depressive symptoms might evolve as a consequence of aberrations within discrete brain networks (rather than in isolated brain regions) that modulate function [22]. Based on the network framework, several neural networks have been proposed to mediate depression disorders, such as the cognitive control network (dorsal prefrontal and parietal regions) [23], the affective network (amygdala, orbitofrontal cortex) [14], and the default mode network (DMN) [24].

A number of methods have been developed for analyzing rs-fMRI data. Independent component analysis (ICA) and region of interest (ROI)-based FC are the two most common approaches used in rs-fMRI studies. ICA is a mathematical method that maximizes statistical independence among its components. Compared with a seed-based approach, ICA has the advantage of requiring few *a priori* assumptions [25]. In addition, ICA can automatically isolate sources of noise; however, it can be difficult to determine whether a component represents physiological noise or a brain network. Thus, the exact separation pattern may be vary between participants [26]. Conversely, the ROI-based FC approach, introduced by Biswal et al [10], is broadly used to measure the temporal synchrony of LFOs among anatomically distributed brain regions [9]. Specifically, in an ROI-based FC analysis, a seed region is *a priori* selection and the subsequent FC map is extracted from the temporal correlations between the ROI and all other voxels in brain or other distributed ROIs [27]. As a method with straightforward statistics and comprehensible results, ROI-based FC analysis has been broadly used in MDD [17], [20], autism spectrum disorders (ASD) [28], and epilepsy [29]. Although ROI-based analysis can provide us with more holistic information regarding a set of brain regions within a network, it is limited by relying on an *a priori* selection [25]. Hence, ROI selection is vital for ROI-based FC analysis, especially for a disorder for which relatively little a priori knowledge of the functionally abnormal brain region has been accumulated. More recently, an approach called ALFF was introduced to detect changes of the BOLD signal in regional spontaneous activity [30]. Unlike the FC approach, ALFF allows observation of the amplitude of spontaneous activity, which is assumed to reflect the absolute intensity of brain spontaneous activity [30]. Furthermore, a modified approach called fractional ALFF (fALFF) has been developed to suppress the physiological noise in the ALFF map [31]. However, the drawback of ALFF and fALFF is that neither method directly reflects the FC [32]. Thus, conducting FC analysis based on ROIs with abnormality revealed by fALFF analysis would lead to insights into the neural basis of nCDSs.

In this study, we sought to explore the alteration of resting-state brain networks in nCDSs by using the ROI-based FC method. Given that little is known about the abnormality of nCDSs, in contrast to previous ROI-based FC studies using *a priori* data [21], [33], we employed the regions with abnormal fALFF as ROIs for further FC analysis. In line with abnormal findings in the depressive disorders mentioned above, we hypothesized that individuals with nCDSs might display aberrant FCs involving a range of brain functional networks.

## Material and Methods

### Participants

From May 2012 to April 2013, a total number of 1105 college students from Guangzhou Medical University were recruited to participate in a survey for the assessment of depressive symptoms. The Beck Depression Inventory (BDI) -IA scale [34], the gold standard of self-rating scales for the assessment of depressive symptom in nonclinical sample, was applied to score depressive severity in the present study's participants. It comprises a total of 21 items, and each answer is scored on a scale ranging from 0 to 3. A BDI score of 10 is the suggested cutoff value for predicting depression [35].

In the present study, subjects with nCDSs were required to have a score of ≥10 on the BDI. Thus, a total of 37 participants were enrolled in rs-fMRI study, including 17 nCDSs subjects with BDI scores from 10 to 32 (5 men and 12 women) and 20 sex-, age-, and education-matched HCs with BDI scores less than 4 (7 men and 13 women). One subject scored >30 on the BDI, indicative of clinical depression but was ruled out by a psychiatrist using DSM IV diagnostic criteria. In addition, all participants enrolled in the rs-fMRI study were required to meet the following criteria: right-handedness, no visualized lesion in any MRI scans, age between 19 and 25, no neurological illness, no alcohol or drug dependence. Demographic data are given in Table 1.

**Table 1:** Demographics and depressive scores of the participants.

Characteristic	nCDSs (n = 17)	HCs (n = 20)	*p* value
Gender (Male/Female)	5/12	7/13	0.498^a^
Age (Mean ± SD Years)	19.06±0.75	19.15±1.03	0.765^b^
Education (Mean ± SD Years)	12.71±0.47	12.95±1.13	0.269^b^
BDI score (Mean ± SD)	15.24±6.73	0.80±1.22	0.000^a^

Abbreviations: nCDSs, non-clinical depressive symptoms; HCs, healthy controls; M, male; F, female; BDI, Beck Depression Inventory; SD, standard deviation.

a and ^b^ indicate the p value for the Chi-Square test and two-sample *t* test respectively.

Written informed consent was obtained from each study participant. The current research protocol was approved by the local Medical Ethics Committee in the affiliated Guangzhou first hospital, Guangzhou Medical University, China.

### Data acquisition

Imaging data were acquired using a 3-Tesla MRI scanner (Siemens, Erlangen, Germany). Participants' head movements were minimized by using foam padding, and headphones were used to reduce scanner noise. Functional images were obtained by using an echo-planar imaging [36] sequence with the following parameters: repetition time (TR)  = 2500 ms, echo time (TE)  = 21 ms, flip angle (FA)  = 90°, field of view (FOV)  = 200 mm×200 mm, matrix  = 64×64, slices  = 42; voxel size  = 3.1 mm×3.1 mm×3.5 mm. The total scanning time was 372 s. In addition, a high-resolution T1-weighted structural image was obtained for each subject using a magnetization-prepared rapid acquisition gradient-echo (MPRAGE) 3-dimensional protocol (TR/TE = 2530 ms/2.34 ms, FA = 7°, FOV = 256 mm×224 mm, slice thickness  = 1.0 mm, skip  = 0 mm). All images were visually inspected to ensure that they did not contain lesions or MRI artifacts before analysis (assessed by a radiologist, Xinhua WEI with 15 years clinical experience). During the entire functional MRI scanning, all participants were asked to relax with their eyes closed but not fall asleep. After scanning, a simple questionnaire was administered to each subject to confirm they had not fallen asleep.

### Imaging data processing and analysis

The resting-state and structural images were preprocessed with the Data Processing Assistant for rs-fMRI (DPARSF, http://www.rest.restfmri.ne) [37] that works with SPM8 (http://www.fil.ion.ucl.ac.uk/spm/software/SPM8) on the Matlab platform. For each subject, the first ten volumes from each run were discarded for signal equilibration. The fMRI images were corrected for the acquisition delay between slices and for the head motion. None of the subjects had more than 2 mm in translation or 2° in rotation and mean point-to-point translation or rotation >0.15 or 0.1° [38]. Then, 3D structural images were registered into standard Montreal Neurological Institute (MNI) space using the unified segmentation DARTEL algorithm [39]. Regression of nuisance was conducted with the signals of white matter and cerebral spinal fluid BOLD-signal, as well as six head-motion profiles to minimize the effects of head motion. After this, registration was completed by applying the functional images to MNI space using the parameters of structural image normalization and with resampling to 3×3×3 mm^3^. Subsequently, the processed images were spatially smoothed with a Gaussian kernel of 4-mm full-width at half maximum (FWHM). Finally, linear trend subtraction and temporal filtering (band pass: 0.01–0.08 Hz) were performed to reduce the effects of low-frequency drift and high frequency noise [10].

FALFF analyses were carried out using the REST package (http://resting-fmri. sourceforge.net) [40]. The calculation procedure has been previously described [31], [41]. Briefly, after the linear trend was removed, the time series for each voxel were transformed to a frequency domain without band-pass filtering. The square root was calculated at each frequency of the power spectrum. The sum of the amplitude across 0.01–0.08 Hz was divided by that across the entire frequency range, i.e., 0–0.25 Hz [31]. To explore the difference in fALFF between the two groups, a two-sample *t*-test was performed in a voxel-by-voxel manner and the age, gender, education, and gray matter volume of each subject were taken as covariates to avoid any undetected effects. A threshold of *p*<0.005 was chosen, corrected by Monte Carlo simulations in the AFNI AlphaSim program (http://afni.nih.gov/afni/docpdf/AlphaSim.pdf). Using this program, clusters that were greater than 12 voxels were applied to the resulting statistical map at a corrected significance level of *p*<0.05.

After the fALFF analysis described above, the peak MNI coordinates, produced from the regions of altered fALFF maps determined via comparison of the two groups, were used as the seed ROIs for a voxel-vise FC analysis comparing between nCDSs and HCs. The FC analysis was conducted with the REST package (REST, http://resting-fmri.sourceforge.net). For each subject, a mean time series for every ROI was computed separately as a reference time course and then correlation analysis was performed between the seed ROI and the remaining voxels in the whole brain. As a result, a correlation map was produced for each seed ROI. The resulting *r* values were transformed into *z* values using Fisher's transformation to improve normality. For each group and seed, individual *z* maps were entered into a voxel wise two-sample *t*-test to determine group differences in FC between the nCDSs and HCs (*p*<0.01, corrected using the same method as in the group of fALFF comparisons).

## Results

### Demographics and depressive scores

There were no significant difference between nCDSs and HCs with regard to gender, age, and year of education. BDI scores were significantly greater in the nCDSs subjects compared to HCs (*p*<0.05) (Table 1).

### Brain regions with altered fALFF

**Figure 1:**
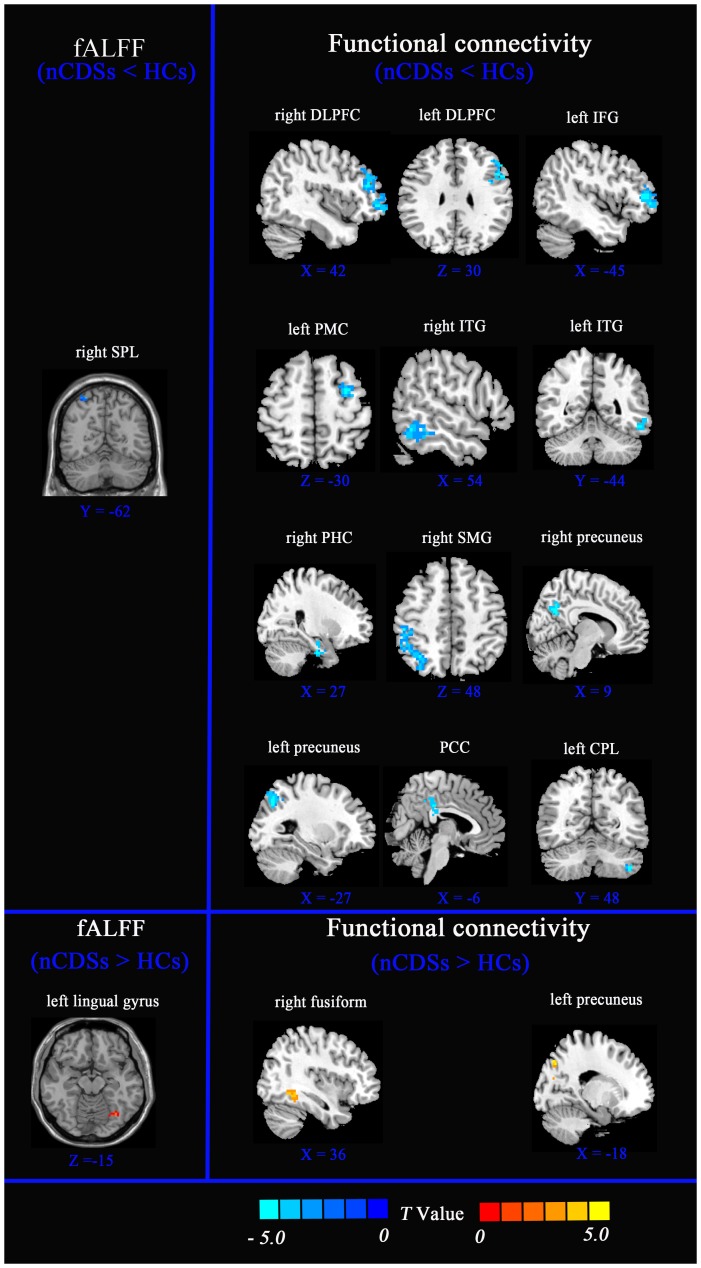
The aberrant fALFF and dysfunctional functional connectivity in nCDSs subjects. Regions showing decreased and increased (red) fALFF (left column) (two-sample t-tests, with threshold at p<0.005, corrected) and functional connectivity (right column) in nCDSs compared to HCs. (two-sample *t*-tests, with threshold at *p*<0.01, corrected). Each altered functional connectivity brain area has the one of coronal, sagittal, or axial views with the MNI location. Color bar indicates the *T* score. SPL, superior parietal lobule superior; DLPFC, dorsolateral prefrontal cortex; IFC, inferior frontal gurus; PMC, premotor cortex; PCC, posterior cingulate cortex; SMG, supramarginal gyrus; PHG, parahippocampal gyrus; ITG, inferior temporal gurus; CPL, left cerebellum posterior lobe

Significant fALFF differences were observed between nCDSs and HCs (*p*<0.005, corrected). Compared with HCs, nCDSs subjects were found to have decreased fALFF in the right superior parietal lobule (SPL) [Broadman's area 7 (BA 7)], and increased fALFF in the left lingual gyrus (BA 19) (Fig. 1 and Table 2).

**Table 2:** Difference of fALFF in Individuals with Nonclinical Depressive Symptoms and Control Subjects.

Brain Region	BA	Cluster Size (voxels)	MNI Coordinates (mm)			*T* Value
			X	Y	Z	
Nonclinical depressive symptoms < Healthy controls						
right superior parietal lobule	7	100	28	−62	63	−4.6451
Nonclinical depressive symptoms > Healthy controls						
left lingual gyrus	19	151	−33	−66	−15	4.5091

fALFF, fractional amplitude of low-frequency fluctuations, BA, Brodman's area; MNI, Montreal Neurological Institute.

### Brian regions with aberrant functional connectivity

Applying the right SPL (BA 7) as the seed ROI and compared with HCs, nCDSs showed reduced FCs in the bilateral dorsal lateral prefrontal cortex (DLPFC)(BA 46), left inferior frontal gurus (IFG)(BA 44), left premotor cortex (PMC) (BA 6), DMN network [i.e., bilateral precuneus (BA 7), posterior cingulate cortex (PCC) (BA 23), right supramarginal gyrus (SMG)(BA 40), right parahippocampal gyrus (PHG) (BA 36), bilateral inferior temporal gurus (ITG) (BA20)] and left cerebellum posterior lobe(CPL). Additionally, using the left lingual gyrus (BA 19) as a seed ROI, increased FCs were found in the right fusiform (BA 37) and left precuneus (BA 7) (Fig. 1 and Table 3).

**Table 3:** Difference of Functional Connectivity in Nonclinical Depressive Symptoms Individuals and Control Subjects.

Seed Area	Connected Brain Region	BA	voxels Size	MNI coordinates (mm)			*T* value
				X	Y	Z	
Nonclinical depressive symptoms < Healthy controls							
right SPL	right DLPFC	46	286	42	51	−3	−5.3407
	left DLPFC	46	135	−45	50	12	−5.147
	left IFG	44	188	−54	12	30	−4.7973
	left PMC	6	111	−30	−3	57	−5.964
	right ITG	20	155	54	−42	−15	−5.3528
	left ITG	20	103	−60	−44	−18	−4.223
	right PHC	36	63	27	−14	−33	−4.5485
	right SMG	40	591	51	−38	48	−5.7587
	right precuneus	7	147	9	−64	36	−4.2006
	left precuneus	7	543	−27	−69	39	−5.8875
	PCC	23	50	−6	21	41	−4.5467
	left CPL	/	64	−45	−48	−51	−4.2939
Nonclinical depressive symptoms > Healthy controls							
left lingual gyrus	right fusiform	37	111	36	−51	−9	6.0675
	left precuneus	7	29	−18	−78	45	4.8091

BA, Brodman's area; MNI, Montreal Neurological Institute; SPL, superior parietal lobule superior; DLPFC, dorsolateral prefrontal cortex; IFC, inferior frontal gurus; PMC, premotor cortex; PCC, posterior cingulate cortex; SMG, supramarginal gyrus; PHG, parahippocampal gyrus; ITG, inferior temporal gurus; CPL, left cerebellum posterior lobe.

## Discussion

The present study provides primary evidence for the neural underpinning of nCDSs. After fALFF analysis, regions of the right SPL and left lingual gyrus with aberrant spontaneous activity were applied as seed ROIs for FC analysis. Compared with HCs, subjects with nCDSs showed reduced FCs between the right SPL and bilateral DLPFC, left IFG, left PMC, and DMN network (i.e., bilateral precuneus, PCC, right SMG, right PHG, bilateral ITG), as well as the left CPL. In addition, increased FCs were seen between the left lingual gyrus and right fusiform as well as in the left precuneus. The aim of this study is to provide novel insight on the underlying mechanism of nCDSs. In contrast to previous studies, we applied areas with altered fALFF comparison between two groups as seed ROIs for further FC analysis. The aim was to overcome the limitation of relying on *a priori* selection in ROI-based FC analysis. To our knowledge, this is the first evidence providing insight into spontaneous activity in nCDSs subjects from a sample of college students.

### Aberrant FCs in right SPL

Decreased fALFF in the right SPL (BA 7) in nCDSs was observed in this study. Moreover, altered FCs were identified between right SPL and several brain regions including bilateral DLPFC, left IFG, left PMC, DMN network (i.e., bilateral precuneus, PCC, right SMG, right PHG, bilateral ITG) and left CPL. The SPL and DLPFC were proposed to be the main components of the executive control network. Anatomical evidence from primates demonstrated that the DLPFC has dense interconnections with the lateral parietal cortex [42]. Moreover, a large body of studies consistently support the DLPFC play a critical role in the dysfunction of emotion adjustment in MDD [43]–[45]. Generally, DLPFC was considered to play the role of “cognitive” or “executive” function [43], such as the manipulation of working memory, intention formation, goal-directed action, and attention control [46]. Thus, we speculate that SPL together with DLPFC constitutes the neural substrate of top-down control processes participating in emotion regulation [47]. Decreased FC was found in the left IFG (BA 44) in this study. In addition to language-related function, the left IFG has been increasingly implicated in emotion recognition tasks [48]. Furthermore, deficits in emotion recognition appeared after damage to the frontal operculum (involving BA 44) [49]. Therefore, we suppose that the dysfunction of emotion recognition may be a possible mechanism of underlying nCDSs. Interestingly, decreased FC between the right SPL and left PMC was identified in the present study. It is generally assumed that emotional processes may affect behavioral responses at the motor preparation stages [50]. Abnormal connectivity in the PMA indicated motor preparation dysfunction in depression patients [51], [52]. Moreover, the motor retardation is a classic feature of depression [51], [53]. Recent rs-fMRI studies also reported altered ReHo in the PMA in late-life subthreshold depression subjects [54]. Thus, considering the decreased FC observed in the PMA in this study, we hypothesize that motor preparation might be slowed in nCDSs. Consistent with results concerning depressive disorder [23], [24], we found decreased FCs in several regions belonging to the DMN. Although the exact functions of this network in humans remains unclear, the DMN is considered to be important for internally focused tasks, such as self-referential thought, autobiographical memory retrieval, future planning, and theory of mind [55], [56]. Abnormal activity and FC related to the DMN were reported in several studies of MDD [57]. Failure of DMN deactivation during emotional or cognitive tasks has been proposed as a possible mechanism acting in depression [24]. In line with these findings, we guess that nCDSs might be involved in a pathological inability of the DMN to regulate self-referential activity in appropriate manner. Additionally, decreased FC between the right SPL and left cerebellum posterior lobe was identified in the present study. The main function of the cerebellum has traditionally been though to involve sensorimotor function. However, several lines of evidence have suggested that the cerebellum may play a role in the regulation of emotion [58] and cognitive processing of negative stimuli [59]. Indeed, the cerebellum connects both cortical and limbic brain regions anatomically [60]. Thus, the aberrant connection between the SPL and cerebellum might be the potential mechanism of nCDSs. On the basis of this evidence, we speculate that the right SPL playing vital role in modulating emotion in nCDSs. The reduced FCs between right SPL and corresponding functional brain regions indicate alternations of attention and cognition, and motor preparation as well as self-referential which are associated with the symptoms of nCDSs.

### Aberrant FCs in the left lingual gyrus

Compared to HCs, we found increased connectivity between the lingual gyrus (BA19) and right fusiform (BA 37) as well as the left precuneus (BA7) in nCDSs. Previous studies have suggested that the lingual gyrus and fusiform gyrus are components of the visual recognition network [61]. Additionally, task-fMRI studies implicated visual cortical regions and the medial PFC, amygdala, and insula in face recognition and emotion decoding [62]. These findings indicate dysfunction of visual recognition resulting in abnormal emotional control related visual information processing in nCDSs. In addition to emotion control, of the literature concerning depression indicates pronounced deficits in a range of cognitive domains [2]. As a core component of DMN [63], [64], the precuneus has been commonly described with aberrant activity or disturbance to functional connectivity in depressive patients [24], [65]. It has been proposed to play a role in visuospatial processing [66] and emotional processing [63]. Moreover, positive connectivity has been observed between the medial dorsal-posterior precuneus and lingual gurus [67]. Taking this evidence together, we hypothesize that increased fALFF in the left lingual gyrus and its increased connectivity with the right fusiform and left precuneus may be a type of compensation regulation for visuospatial processing.

### Limitations

Several limitations of this study should be mentioned. First, the participants were limited to young adults from the same college, maximizing the consistency with regard to age, education age and living condition between subjects. However, the results of this study should be verified in a diverse population. Second, the sample size in this study was relatively small, and we did not divide the subjects into more subgroups of nCDSs based on BDI scores. As a result, a compound effect of the different degrees of depressive symptoms could not be avoided. Owing to the relatively small sample size, we could not find fALFF abnormalities in the prefrontal and subcortical affective networks. Future studies with a larger sample size are necessary to affirm the results. Third, the fALFF and FC approaches in this study were based on the same dataset. Regions showing altered fALFF were further used to study FC, which potentially increases the chance of finding FC differences as local fALFF alterations are likely associated with altered FC patterns. Finally, nCDSs subjects have relatively mild depressive symptoms that may transform over time. Therefore, a longitudinal study is needed to validate the present results.

## Conclusion

By applying the fALFF results to FC analysis, we found that subjects with nCDSs have altered spontaneous neuronal activity in regions including the right SPL and left lingual gyrus, as well as altered brain circuits implicated in attentional, cognitive and emotional function. Our findings provide novel and important insights into the underlying neural mechanism of nCDSs. Our results have provided neural evidences of preventive intervention for the subjects with nCDSs. Future research will be focused on neural basis of progression of subclinical depressive symptoms to clinical depression.

## References

[1] Jamison DT, Breman JG, Measham AR, Alleyne G, Claeson M, et al**.** (2006). Priorities in Health: Disease Control Priorities Companion Volume. Washington DC: The International Bank for Reconstruction and Development/The World Bank Group. 21089239

[2] Kerestes R, Davey CG, Stephanou K, Whittle S, Harrison BJ (2013) Functional brain imaging studies of youth depression: A systematic review. Neuroimage Clin 4:209–231. 24455472 10.1016/j.nicl.2013.11.009 PMC3895619

[3] Mikolajczyk RT, Maxwell AE, Naydenova V, Meier S, El Ansari W (2008) Depressive symptoms and perceived burdens related to being a student: Survey in three European countries. Clin Pract Epidemiol Ment Health 4:19. 18598340 10.1186/1745-0179-4-19 PMC2483702

[4] Mikolajczyk RT, Maxwell AE, El Ansari W, Naydenova V, Stock C, et al (2008) Prevalence of depressive symptoms in university students from Germany, Denmark, Poland and Bulgaria. Soc Psychiatry Psychiatr Epidemiol 43:105–112. 18038173 10.1007/s00127-007-0282-0

[5] Al-Busaidi Z, Bhargava K, Al-Ismaily A, Al-Lawati H, Al-Kindi R, et al (2011) Prevalence of Depressive Symptoms among University Students in Oman. Oman Med J 26:235–239. 22043426 10.5001/omj.2011.58 PMC3191716

[6] Melo-Carrillo A, Van Oudenhove L, Lopez-Avila A (2012) Depressive symptoms among Mexican medical students: high prevalence and the effect of a group psychoeducation intervention. J Affect Disord 136:1098–1103. 22119092 10.1016/j.jad.2011.10.040

[7] Blazer DG, Kessler RC, McGonagle KA, Swartz MS (1994) The prevalence and distribution of major depression in a national community sample: the National Comorbidity Survey. Am J Psychiatry 151:979–986. 8010383 10.1176/ajp.151.7.979

[8] Georgiades K, Lewinsohn PM, Monroe SM, Seeley JR (2006) Major depressive disorder in adolescence: the role of subthreshold symptoms. J Am Acad Child Adolesc Psychiatry 45:936–944. 16865036 10.1097/01.chi.0000223313.25536.47

[9] Fox MD, Raichle ME (2007) Spontaneous fluctuations in brain activity observed with functional magnetic resonance imaging. Nat Rev Neurosci 8:700–711. 17704812 10.1038/nrn2201

[10] Biswal B, Yetkin FZ, Haughton VM, Hyde JS (1995) Functional connectivity in the motor cortex of resting human brain using echo-planar MRI. Magn Reson Med 34:537–541. 8524021 10.1002/mrm.1910340409

[11] Wang L, LaBar KS, Smoski M, Rosenthal MZ, Dolcos F, et al (2008) Prefrontal mechanisms for executive control over emotional distraction are altered in major depression. Psychiatry Res 163:143–155. 18455373 10.1016/j.pscychresns.2007.10.004 PMC2553159

[12] Ye T, Peng J, Nie B, Gao J, Liu J, et al (2012) Altered functional connectivity of the dorsolateral prefrontal cortex in first-episode patients with major depressive disorder. Eur J Radiol 81:4035–4040. 22939367 10.1016/j.ejrad.2011.04.058

[13] Greicius MD, Flores BH, Menon V, Glover GH, Solvason HB, et al (2007) Resting-state functional connectivity in major depression: abnormally increased contributions from subgenual cingulate cortex and thalamus. Biol Psychiatry 62:429–437. 17210143 10.1016/j.biopsych.2006.09.020 PMC2001244

[14] Zhang X, Zhu X, Wang X, Zhong M, Yi J, et al (2014) First-episode medication-naive major depressive disorder is associated with altered resting brain function in the affective network. PLoS One 9:e85241. 24416367 10.1371/journal.pone.0085241 PMC3887023

[15] Liu CH, Ma X, Wu X, Li F, Zhang Y, et al (2012) Resting-state abnormal baseline brain activity in unipolar and bipolar depression. Neurosci Lett 516:202–206. 22503728 10.1016/j.neulet.2012.03.083

[16] Guo WB, Liu F, Xue ZM, Xu XJ, Wu RR, et al (2012) Alterations of the amplitude of low-frequency fluctuations in treatment-resistant and treatment-response depression: a resting-state fMRI study. Prog Neuropsychopharmacol Biol Psychiatry 37:153–160. 22306865 10.1016/j.pnpbp.2012.01.011

[17] Anand A, Li Y, Wang Y, Lowe MJ, Dzemidzic M (2009) Resting state corticolimbic connectivity abnormalities in unmedicated bipolar disorder and unipolar depression. Psychiatry Res 171:189–198. 19230623 10.1016/j.pscychresns.2008.03.012 PMC3001251

[18] Anand A, Li Y, Wang Y, Wu J, Gao S, et al (2005) Activity and connectivity of brain mood regulating circuit in depression: a functional magnetic resonance study. Biol Psychiatry 57:1079–1088. 15866546 10.1016/j.biopsych.2005.02.021

[19] Anand A, Li Y, Wang Y, Gardner K, Lowe MJ (2007) Reciprocal effects of antidepressant treatment on activity and connectivity of the mood regulating circuit: an FMRI study. J Neuropsychiatry Clin Neurosci 19:274–282. 17827412 10.1176/appi.neuropsych.19.3.274 PMC3465666

[20] Cullen KR, Gee DG, Klimes-Dougan B, Gabbay V, Hulvershorn L, et al (2009) A preliminary study of functional connectivity in comorbid adolescent depression. Neurosci Lett 460:227–231. 19446602 10.1016/j.neulet.2009.05.022 PMC2713606

[21] Lui S, Wu Q, Qiu L, Yang X, Kuang W, et al (2011) Resting-state functional connectivity in treatment-resistant depression. Am J Psychiatry 168:642–648. 21362744 10.1176/appi.ajp.2010.10101419

[22] Wang L, Hermens DF, Hickie IB, Lagopoulos J (2012) A systematic review of resting-state functional-MRI studies in major depression. J Affect Disord 142:6–12. 22858266 10.1016/j.jad.2012.04.013

[23] Sheline YI, Price JL, Yan Z, Mintun MA (2010) Resting-state functional MRI in depression unmasks increased connectivity between networks via the dorsal nexus. Proc Natl Acad Sci U S A 107:11020–11025. 20534464 10.1073/pnas.1000446107 PMC2890754

[24] Sheline YI, Barch DM, Price JL, Rundle MM, Vaishnavi SN, et al (2009) The default mode network and self-referential processes in depression. Proc Natl Acad Sci U S A 106:1942–1947. 19171889 10.1073/pnas.0812686106 PMC2631078

[25] Lee MH, Smyser CD, Shimony JS (2012) Resting-State fMRI: A Review of Methods and Clinical Applications. AJNR Am J Neuroradiol. 10.3174/ajnr.A3263 PMC4035703 22936095

[26] Rosazza C, Minati L, Ghielmetti F, Mandelli ML, Bruzzone MG (2012) Functional connectivity during resting-state functional MR imaging: study of the correspondence between independent component analysis and region-of-interest-based methods. AJNR Am J Neuroradiol 33:180–187. 21998099 10.3174/ajnr.A2733 PMC7966157

[27] Margulies DS, Bottger J, Long X, Lv Y, Kelly C, et al (2010) Resting developments: a review of fMRI post-processing methodologies for spontaneous brain activity. MAGMA 23:289–307. 20972883 10.1007/s10334-010-0228-5

[28] Weng SJ, Wiggins JL, Peltier SJ, Carrasco M, Risi S, et al (2010) Alterations of resting state functional connectivity in the default network in adolescents with autism spectrum disorders. Brain Res 1313:202–214. 20004180 10.1016/j.brainres.2009.11.057 PMC2818723

[29] Luo C, Li Q, Lai Y, Xia Y, Qin Y, et al (2011) Altered functional connectivity in default mode network in absence epilepsy: a resting-state fMRI study. Hum Brain Mapp 32:438–449. 21319269 10.1002/hbm.21034 PMC6870112

[30] Zang YF, He Y, Zhu CZ, Cao QJ, Sui MQ, et al (2007) Altered baseline brain activity in children with ADHD revealed by resting-state functional MRI. Brain Dev 29:83–91. 16919409 10.1016/j.braindev.2006.07.002

[31] Zou QH, Zhu CZ, Yang Y, Zuo XN, Long XY, et al (2008) An improved approach to detection of amplitude of low-frequency fluctuation (ALFF) for resting-state fMRI: fractional ALFF. J Neurosci Methods 172:137–141. 18501969 10.1016/j.jneumeth.2008.04.012 PMC3902859

[32] Vargas C, Lopez-Jaramillo C, Vieta E (2013) A systematic literature review of resting state network-functional MRI in bipolar disorder. J Affect Disord 150:727–735. 23830141 10.1016/j.jad.2013.05.083

[33] Tahmasian M, Knight DC, Manoliu A, Schwerthoffer D, Scherr M, et al (2013) Aberrant intrinsic connectivity of hippocampus and amygdala overlap in the fronto-insular and dorsomedial-prefrontal cortex in major depressive disorder. Front Hum Neurosci 7:639. 24101900 10.3389/fnhum.2013.00639 PMC3787329

[34] Beck AT, Ward CH, Mendelson M, Mock J, Erbaugh J (1961) An inventory for measuring depression. Arch Gen Psychiatry 4:561–571. 13688369 10.1001/archpsyc.1961.01710120031004

[35] Ignjatovic-Ristic D, Hinic D, Jovic J (2012) Evaluation of the Beck Depression Inventory in a nonclinical student sample. West Indian Med J 61:489–493. 23441370 10.7727/wimj.2011.215

[36] Bandelow B, Zohar J, Hollander E, Kasper S, Moller HJ, et al (2008) World Federation of Societies of Biological Psychiatry (WFSBP) guidelines for the pharmacological treatment of anxiety, obsessive-compulsive and post-traumatic stress disorders - first revision. World J Biol Psychiatry 9:248–312. 18949648 10.1080/15622970802465807

[37] Yan CG, Zang YF (2010) DPARSF: A MATLAB Toolbox for "Pipeline" Data Analysis of Resting-State fMRI. Front Syst Neurosci 4:13. 20577591 10.3389/fnsys.2010.00013 PMC2889691

[38] Van Dijk KR, Sabuncu MR, Buckner RL (2012) The influence of head motion on intrinsic functional connectivity MRI. Neuroimage 59:431–438. 21810475 10.1016/j.neuroimage.2011.07.044 PMC3683830

[39] Bergouignan L, Chupin M, Czechowska Y, Kinkingnehun S, Lemogne C, et al (2009) Can voxel based morphometry, manual segmentation and automated segmentation equally detect hippocampal volume differences in acute depression? Neuroimage 45:29–37. 19071222 10.1016/j.neuroimage.2008.11.006

[40] Song XW, Dong ZY, Long XY, Li SF, Zuo XN, et al (2011) REST: a toolkit for resting-state functional magnetic resonance imaging data processing. PLoS One 6:e25031. 21949842 10.1371/journal.pone.0025031 PMC3176805

[41] Turner JA, Damaraju E, van Erp TG, Mathalon DH, Ford JM, et al (2013) A multi-site resting state fMRI study on the amplitude of low frequency fluctuations in schizophrenia. Front Neurosci 7:137. 23964193 10.3389/fnins.2013.00137 PMC3737471

[42] Barbas H (2000) Connections underlying the synthesis of cognition, memory, and emotion in primate prefrontal cortices. Brain Res Bull 52:319–330. 10922509 10.1016/s0361-9230(99)00245-2

[43] Koenigs M, Grafman J (2009) The functional neuroanatomy of depression: distinct roles for ventromedial and dorsolateral prefrontal cortex. Behav Brain Res 201:239–243. 19428640 10.1016/j.bbr.2009.03.004 PMC2680780

[44] Phillips ML, Drevets WC, Rauch SL, Lane R (2003) Neurobiology of emotion perception II: implications for major psychiatric disorders. Biol Psychiatry 54:515–528. 12946880 10.1016/s0006-3223(03)00171-9

[45] Fitzgerald PB, Oxley TJ, Laird AR, Kulkarni J, Egan GF, et al (2006) An analysis of functional neuroimaging studies of dorsolateral prefrontal cortical activity in depression. Psychiatry Res 148:33–45. 17029760 10.1016/j.pscychresns.2006.04.006

[46] Miller EK, Cohen JD (2001) An integrative theory of prefrontal cortex function. Annu Rev Neurosci 24:167–202. 11283309 10.1146/annurev.neuro.24.1.167

[47] Viviani R (2013) Emotion regulation, attention to emotion, and the ventral attentional network. Front Hum Neurosci 7:746. 24223546 10.3389/fnhum.2013.00746 PMC3819767

[48] Wildgruber D, Riecker A, Hertrich I, Erb M, Grodd W, et al (2005) Identification of emotional intonation evaluated by fMRI. Neuroimage 24:1233–1241. 15670701 10.1016/j.neuroimage.2004.10.034

[49] Adolphs R, Baron-Cohen S, Tranel D (2002) Impaired recognition of social emotions following amygdala damage. J Cogn Neurosci 14:1264–1274. 12495531 10.1162/089892902760807258

[50] Williams JM, Mathews A, MacLeod C (1996) The emotional Stroop task and psychopathology. Psychol Bull 120:3–24. 8711015 10.1037/0033-2909.120.1.3

[51] Liberg B, Adler M, Jonsson T, Landen M, Rahm C, et al (2013) Motor imagery in bipolar depression with slowed movement. J Nerv Ment Dis 201:885–893. 24080676 10.1097/NMD.0b013e3182a5c2a7

[52] Rogers MA, Bradshaw JL, Phillips JG, Chiu E, Mileshkin C, et al (2002) Mental rotation in unipolar major depression. J Clin Exp Neuropsychol 24:101–106. 11935428 10.1076/jcen.24.1.101.974

[53] Walther S, Hofle O, Federspiel A, Horn H, Hugli S, et al (2012) Neural correlates of disbalanced motor control in major depression. J Affect Disord 136:124–133. 21930304 10.1016/j.jad.2011.08.020

[54] Ma Z, Li R, Yu J, He Y, Li J (2013) Alterations in regional homogeneity of spontaneous brain activity in late-life subthreshold depression. PLoS One 8:e53148. 23301035 10.1371/journal.pone.0053148 PMC3534624

[55] Buckner RL, Andrews-Hanna JR, Schacter DL (2008) The brain's default network: anatomy, function, and relevance to disease. Ann N Y Acad Sci 1124:1–38. 18400922 10.1196/annals.1440.011

[56] Hoff GE, Van den Heuvel MP, Benders MJ, Kersbergen KJ, De Vries LS (2013) On development of functional brain connectivity in the young brain. Front Hum Neurosci 7:650. 24115929 10.3389/fnhum.2013.00650 PMC3792361

[57] Tadayonnejad R, Ajilore O (2014) Brain network dysfunction in late-life depression: a literature review. J Geriatr Psychiatry Neurol 27:5–12. 24381233 10.1177/0891988713516539

[58] Schutter DJ, van Honk J (2009) The cerebellum in emotion regulation: a repetitive transcranial magnetic stimulation study. Cerebellum 8:28–34. 18855096 10.1007/s12311-008-0056-6

[59] Schraa-Tam CK, Rietdijk WJ, Verbeke WJ, Dietvorst RC, van den Berg WE, et al (2012) fMRI activities in the emotional cerebellum: a preference for negative stimuli and goal-directed behavior. Cerebellum 11:233–245. 21761197 10.1007/s12311-011-0301-2 PMC3311856

[60] Turner BM, Paradiso S, Marvel CL, Pierson R, Boles Ponto LL, et al (2007) The cerebellum and emotional experience. Neuropsychologia 45:1331–1341. 17123557 10.1016/j.neuropsychologia.2006.09.023 PMC1868674

[61] Tao H, Guo S, Ge T, Kendrick KM, Xue Z, et al (2013) Depression uncouples brain hate circuit. Mol Psychiatry 18:101–111. 21968929 10.1038/mp.2011.127 PMC3526729

[62] Haxby JV, Hoffman EA, Gobbini MI (2002) Human neural systems for face recognition and social communication. Biol Psychiatry 51:59–67. 11801231 10.1016/s0006-3223(01)01330-0

[63] Broyd SJ, Demanuele C, Debener S, Helps SK, James CJ, et al (2009) Default-mode brain dysfunction in mental disorders: a systematic review. Neurosci Biobehav Rev 33:279–296. 18824195 10.1016/j.neubiorev.2008.09.002

[64] Utevsky AV, Smith DV, Huettel SA (2014) Precuneus is a functional core of the default-mode network. J Neurosci 34:932–940. 24431451 10.1523/JNEUROSCI.4227-13.2014 PMC3891968

[65] Liu F, Hu M, Wang S, Guo W, Zhao J, et al (2012) Abnormal regional spontaneous neural activity in first-episode, treatment-naive patients with late-life depression: a resting-state fMRI study. Prog Neuropsychopharmacol Biol Psychiatry 39:326–331. 22796277 10.1016/j.pnpbp.2012.07.004

[66] Wenderoth N, Debaere F, Sunaert S, Swinnen SP (2005) The role of anterior cingulate cortex and precuneus in the coordination of motor behaviour. Eur J Neurosci 22:235–246. 16029213 10.1111/j.1460-9568.2005.04176.x

[67] Zhang S, Li CS (2012) Functional connectivity mapping of the human precuneus by resting state fMRI. Neuroimage 59:3548–3562. 22116037 10.1016/j.neuroimage.2011.11.023 PMC3288461

